# Assessing the Risk of a Canine Rabies Incursion in Northern Australia

**DOI:** 10.3389/fvets.2017.00141

**Published:** 2017-08-31

**Authors:** Emily G. Hudson, Victoria J. Brookes, Michael P. Ward

**Affiliations:** ^1^Sydney School of Veterinary Science, The University of Sydney, Camden, NSW Australia

**Keywords:** risk, rabies, Australia, dogs, surveillance

## Abstract

Rabies is a globally distributed virus that causes approximately 60,00 human deaths annually with >99% of cases caused by dog bites. Australia is currently canine rabies free. However, the recent eastward spread of rabies in the Indonesian archipelago has increased the probability of rabies entry into northern Australian communities. In addition, many northern Australian communities have large populations of free-roaming dogs, capable of maintaining rabies should an incursion occur. A risk assessment of rabies entry and transmission into these communities is needed to target control and surveillance measures. Illegal transportation of rabies-infected dogs *via* boat landings is a high-risk entry pathway and was the focus of the current study. A quantitative, stochastic, risk assessment model was developed to evaluate the risk of rabies entry into north-west Cape York Peninsula, Australia, and rabies introduction to resident dogs in one of the communities *via* transport of rabies-infected dogs on illegal Indonesian fishing boats. Parameter distributions were derived from expert opinion, literature, and analysis of field studies. The estimated median probability of rabies entry into north-west Cape York Peninsula and into Seisia from individual fishing boats was 1.9 × 10^−4^/boat and 8.7 × 10^−6^/boat, respectively. The estimated annual probability that at least one rabies-infected dog enters north-west Cape York Peninsula and into Seisia was 5.5 × 10^−3^ and 3.5 × 10^−4^, respectively. The estimated median probability of rabies introduction into Seisia was 4.7 × 10^−8^/boat, and the estimated annual probability that at least one rabies-infected dog causes rabies transmission in a resident Seisia dog was 8.3 × 10^−5^. Sensitivity analysis using the Sobol method highlighted some parameters as influential, including but not limited to the prevalence of rabies in Indonesia, the probability of a dog on board an Indonesian fishing boat, and the probability of a Seisia dog being on the beach. Overall, the probabilities of rabies entry into north-west Cape York Peninsula and rabies introduction into Seisia are low. However, the potential devastating consequences of a rabies incursion in this region make this a non-negligible risk.

## Introduction

Rabies is a vaccine-preventable viral infection that is globally distributed and causes approximately 59,000 human deaths annually (1). Although all mammals can be infected by rabies, domestic dogs are responsible for >99% of human cases (2). In recent years, rabies has spread into rabies-free regions of the world such as areas in Indonesia (3, 4), Bhutan (5, 6), and China (7, 8). Spread has predominately been attributed to transportation of rabies-infected dogs. Effective surveillance is critical for timely detection of rabies incursions. Detection time can subsequently affect the outcome of a rabies incursion from elimination if detected early to endemic infection following delayed detection (9). Surveillance and biosecurity measures (for example, quarantine, import restrictions, or coastal patrols) are also important in rabies-free regions to prevent potential rabies incursions. However, with limited resources, surveillance efforts and control measures must be targeted to areas that have the greatest effect on limiting rabies entry and transmission (10); this is also likely to be cost-efficient.

Risk assessment and subsequent sensitivity analysis are useful tools to assess the likelihood of disease incursions and direct surveillance efforts and control measures (10). There are several recent examples of such studies, in which the risk of entry of rabies *via* legal routes that are controlled by import restrictions have been assessed. For example, a rabies entry risk assessment for the United Kingdom found that a change in importation policy from the current UK Pet Travel Scheme (PETS) to the European Pet Movement Policy would result in a 60-fold increase in rabies entry risk. Sensitivity analysis demonstrated that non-compliance with control measures was a highly influential parameter (11). Non-compliance with control measures—such as improper vaccination or serological protocol and document forgery—has also been found to be influential in earlier assessments of the risk of rabies entry associated with the PETS scheme (12, 13). Another risk assessment of rabies entry into Taiwan also highlighted non-compliance as an influential parameter (14). It has been suggested that illegal smuggling is likely to pose a higher risk of rabies incursion compared to legal modes of transport; in some cases, if illegal smuggling is not reduced to almost zero, the total risk of rabies entry cannot be lowered to an acceptable level (14). Illegal transportation of infected dogs *via* boat has already been responsible for the spread of rabies into previously rabies-free regions of Indonesia such as Flores and Bali (4, 15, 16) and is considered to be a high-risk entry pathway of rabies into other rabies-free areas in the region (17, 18).

Australia is currently canine rabies free. However, the eastward spread of rabies through the Indonesian archipelago has reduced the distance between northern Australian regions, such as the Northern Peninsula Area (NPA) and rabies-infected islands in Indonesia (3). There are cultural connections between Indonesia, Papua New Guinea (PNG), the Torres Strait, and northern mainland Australia, which subsequently cause human movements between the four areas. These movements increase the probability of a rabies incursion into northern Australian communities such as the NPA. In addition, the coastline of northern Australia is vast—approximately 10,000 km between Broome in the west and Cairns in the east (www.agriculture.gov.au/biosecurity/australia/naqs)—and is sparsely populated. Surveillance of the entire region is difficult, which provides opportunity for unregulated boat landings. Deriving parameters for risk assessment of illegal routes of entry is challenging due to the covert nature of illegal activities and subsequent lack of published information. Structured methods to elicit parameters from experts with local knowledge is one source that can be used to provide information for inputs with unknown values (19, 20). In areas in which there are communities such as the NPA, there is also opportunity for rabies establishment following entry due to the large population of free-roaming domestic dogs that reside within such communities (21). Risk assessments that include exposure and transmission of rabies to resident dogs can highlight postborder control areas to minimize the risk of rabies establishment, following an incursion. For example, the probability of dog confinement following entry of an infected dog was an influential parameter in a risk assessment model for rabies introduction into Lombok, Indonesia, and it was suggested that it could be targeted to minimize rabies transmission risk (22). Similarly, a risk assessment for rabies establishment risk in the port of Hokkaido, Japan, from illegal landings of Russian fishing boats found that the probability of contact with wildlife was a highly influential factor and could be targeted to minimize the predicted risk (23).

The risk of rabies entry and transmission to resident dogs in the NPA has not been previously estimated. In this study, we identified potential illegal routes of entry using qualitative methods and then used a quantitative risk assessment model to evaluate the risk of rabies entry into the NPA and rabies entry and transmission to resident dogs in a community in the NPA. The model used parameter estimates derived during a workshop using structured methods to elicit expert opinion, as well as information from field data and literature. A sensitivity analysis was conducted to identify factors that influenced rabies risk. The findings from this study will be used to direct surveillance and control measures to mitigate the risk of rabies introduction in this region.

## Materials and Methods

### Definition of Risk Pathways

Structured interviews to define possible risk pathways for entry of a rabies-infected dog into Cape York Peninsula (direct from Indonesia or *via* PNG and the Torres Strait) were conducted in August 2016 prior to an expert opinion workshop. Biosecurity officers and Land and Sea Rangers (*n* = 8) were purposefully selected as key informants due to their local knowledge and collectively extensive experience of biosecurity-associated activities in the Cape York Peninsula region. Risk pathways considered in the interviews included the mode of transport (boat or plane), direct or indirect travel (from Indonesia or through PNG and the Torres Strait), reason for travel, and landing sites in the Cape York Peninsula. The highest risk pathways for further investigation using quantitative methods were selected based on agreement between key informants.

**Figure 1:**
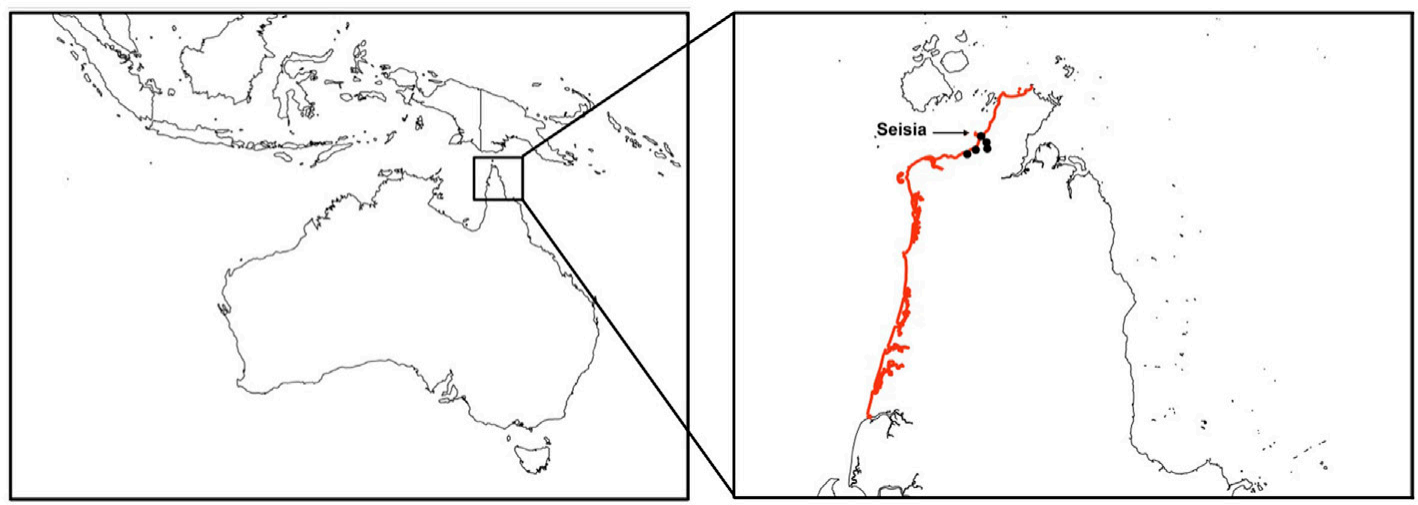
Cape York Peninsula, Australia, with the region of entry of Indonesian fishing boats considered in a risk assessment of the entry of a rabies-infected dog highlighted. This region includes the five communities of the Northern Peninsula Area. The community of Seisia is labeled.

Key informants confirmed that illegal boats were the likely mode of transport of dogs from Indonesia to Cape York Peninsula. The main reason for travel of Indonesian people was believed to be fishing, specifically for shark-fin. The main reasons for a dog on board a fishing boat was for hunting and companionship. They also stated that these boats travel directly from Indonesia to Northern Australia and arrive along the Cape York Peninsula coast and do not travel *via* PNG and the Torres Strait. Informants described that likely locations of entry were in remote areas along the north-western coastline of Cape York Peninsula between the northern section of the NPA (10.6693° 142.533° E) and south to the mouth of the Skardon River (11.757° S 142.002° E; a distance of approximately 122 km; Figure 1). This area is subsequently referred to as north-west Cape York Peninsula. Informants stated that the likely entry point of Indonesian boats specifically into the NPA communities was at Seisia.

Two scenarios were selected for quantitative risk assessment: *Scenario 1*—the entry of a rabies-infected dog to north-west Cape York Peninsula (including the NPA) *via* an illegal fishing boat from Indonesia, which stays for a few days; *Scenario 2*—exposure and infection of a resident, domestic dog following entry of a rabies-infected dog to the beach community of Seisia.

We considered the following pathway for *Scenario 1*. An illegal fishing boat leaves Indonesia and is carrying at least one rabies-infected dog. The infected dog (or dogs) survives the journey to Australia and disembarks the boat. Exposure and transmission to a resident dog is not included in this scenario. *Scenario 2* refines and extends *Scenario 1*; an illegal Indonesian fishing boat traveling to north-west Cape York Peninsula lands briefly on the beach at Seisia (Figure 1). An Indonesian, rabies-infected dog disembarks the boat and comes into effective contact with at least one resident, domestic dog that subsequently develops rabies. The boat and the Indonesian dog leave Seisia.

**Figure 2:**
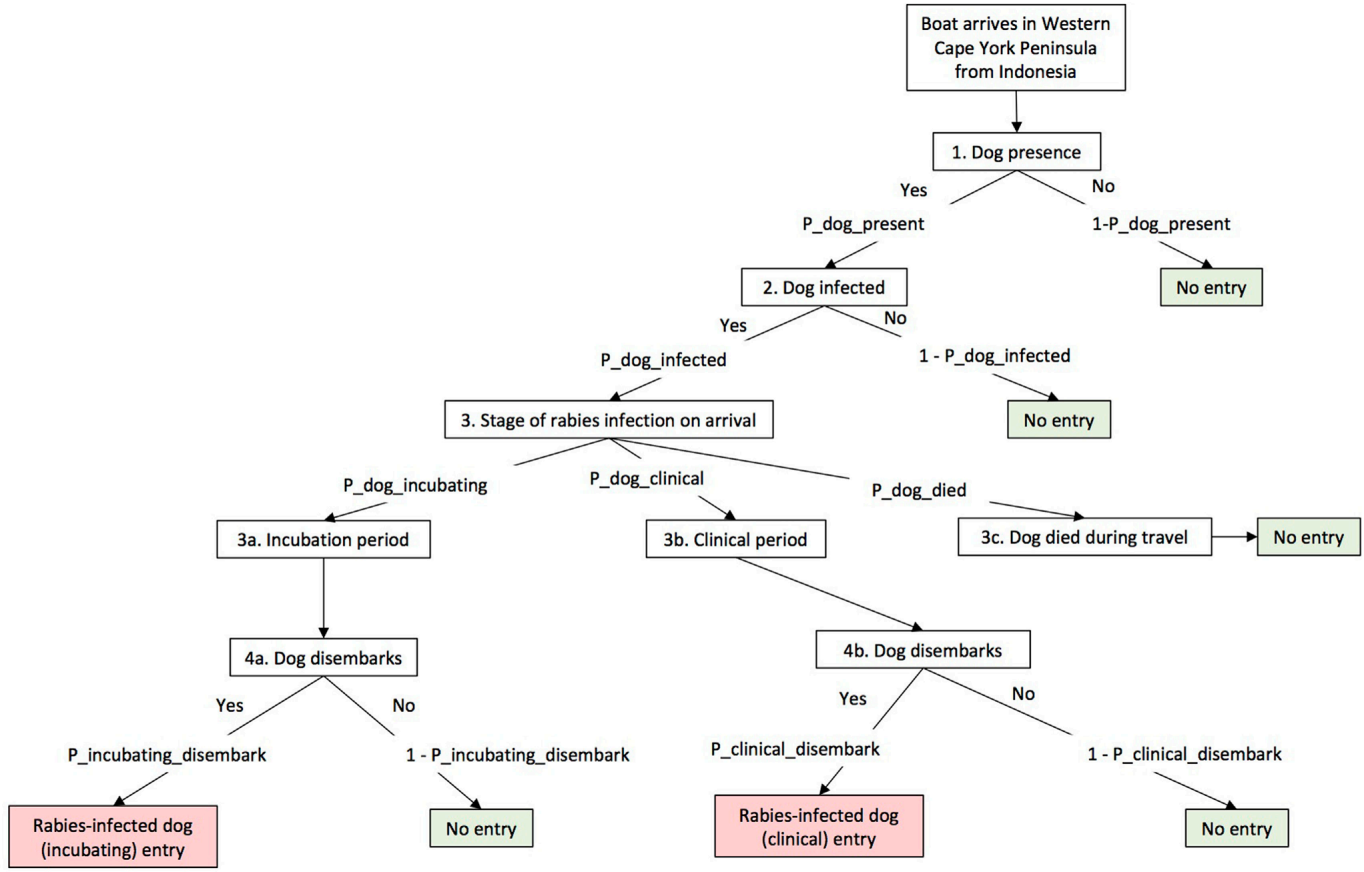
Scenario tree illustrating entry pathway of rabies into north-west Cape York Peninsula, Australia, used in this risk assessment model.

**Figure 3:**
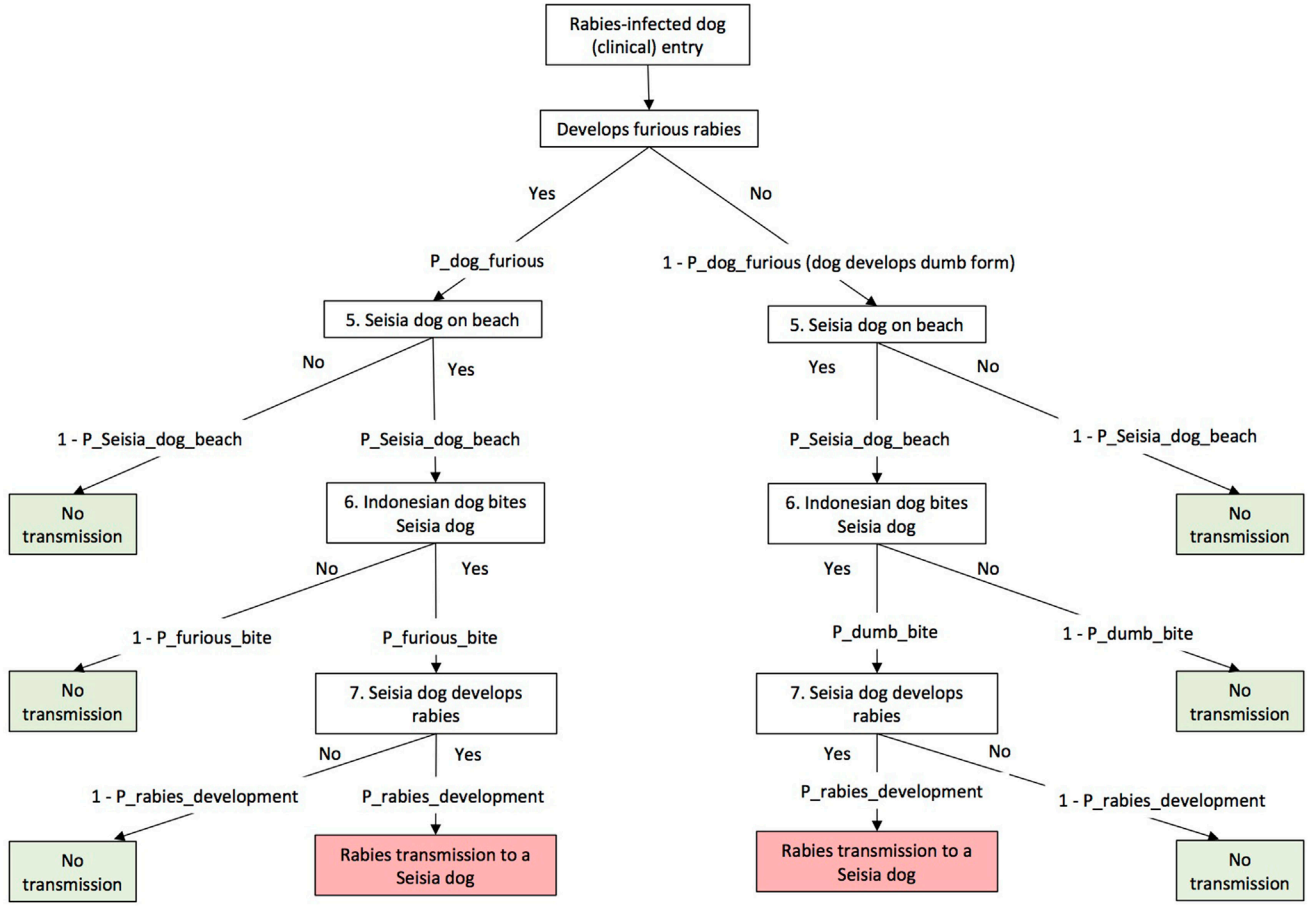
Scenario trees illustrating exposure pathways of rabies after entry into Seisia used in this risk assessment model.

By using the generic scenario tree developed by Ward and Hernández-Jover (10) as a framework, a scenario tree was developed to describe the pathways (Figures 2 and 3). Nodes 1–4 relate to the entry assessment into north-west Cape York Peninsula (including Seisia), and Nodes 5–7 relate to the assessment of rabies exposure and subsequent transmission a dog in Seisia (*Scenario 2*). The nodes were described using 22 parameters of which 8 were derived from the literature, 12 from an expert opinion workshop conducted in August 2016, and 2 from the analysis of field data. Assumptions, nodes, and parameters are described in detail below and in Table 1. Quantitative, stochastic, risk assessment models were developed to evaluate these pathways. Model outputs are described below. The sensitivity of these outputs to model inputs was assessed using the Sobol method (also described below).

**Table 1:** List of model parameters and their values for a risk assessment of canine rabies entry *via* Indonesian fishing boats into north-west Cape York Peninsula and rabies introduction to a resident dog in Seisia, Queensland, Australia.

Node	Parameter	Description	Value	Source
1	Probability boat carrying at least one dog	PERT (min, most likely, max)	0.0217, 0.1196, 0.4329	Expert opinion
2	Prevalence of rabies in Indonesia (0—1)	Uniform (min, max)	0.0001, 0.05	Kitala et al. (24); Cleaveland et al. (25); Kayali et al. (26); Tenzin et al. (5); Tenzin et al. (6); Mustiana (22); Putra et al. (4)
3	Incubation period (days)	Lognormal (mean, SD)	23.6, 17.3	Tojinbara et al. (27)
	Clinical period (days)	Gamma (shape, rate)	2.83, 0.91	Hampson et al. (28)
	Duration of travel (days)	Empirical (min, median, max)	1.092, 4.711, 24.61	Expert opinion
	Probability arrives in incubation period	Empirical (min, median, max)	0.6386, 0.6528, 0.6715	Derived from expert opinion parameters
	Probability arrives in clinical period	Empirical (min, median, max)	0.1548, 0.1708, 0.1809	Derived from expert opinion parameters
	Probability dies during travel	Empirical (min, median, max)	0.1654, 0.1764, 0.1902	Derived from expert opinion parameters
4	Duration of stay (days)	Empirical (min, median, max)	0.956, 5.177, 109.7	Expert opinion
	Probability apparently healthy dog disembarks <5 days stay	PERT (min, most likely, max)	0.0213, 0.0851, 0.1	Expert opinion
	Probability apparently healthy dogs disembarks ≥5 days stay	PERT (min, most likely, max)	0.0213, 0.0851, 0.319	Expert opinion
	Combined probability apparently healthy dog disembarks	Empirical (min, median, max)	0.023, 0.08545, 0.300	Derived from expert opinion parameters
	Probability a clinically affected dog disembarks	PERT (min, most likely, max)	0.021, 0.021, 0.051	Expert opinion
5	Probability a boat lands on Seisia beach	Point value	0.0455	Expert opinion
	Total daily beach-minutes (minutes)	Empirical (min, median, max)	141, 379, 7,710	Field data
	Daily probability that a resident dog is on Seisia beach	Empirical (min, median, max)	0.089, 0.264, 0.556	Field data
6	Probability dog develops furious rabies	Uniform (min, max)	0.1, 0.6	Vaughn et al. (29); Fekadu and Shaddock (30); Foggin (31)
	Probability dog develops dumb rabies	Uniform (min, max)	1—Uniform (0.1, 0.6)	n/a
	Probability of dog bite if dumb form	Uniform (min, max)	0.001, 0.1	Field data
	Probability of dog bite if furious form	Uniform (min, max)	0.5, 0.8	Hampson et al. (28)
7	Probability of rabies development after bite	Uniform (min, max)	0.35, 0.52	Hampson et al. (28)
8	Number of boats that arrive the north-west Cape York Peninsula/year	Empirical (min, median, max)	1, 6, 23	Expert opinion
	Number of boats that arrive in the NPA (Seisia)/year	Empirical (min, median, max)	0.0455 × empirical (1, 6, 23)	Derived from expert opinion parameters
	Number of dogs per boat	Empirical (min, median, max)	1, 2, 5	Expert opinion

*Probabilities are given in the range 0–1*.

### Model Outputs

The following model outputs were derived using Monte Carlo simulation (10,000 iterations) of quantitative, stochastic models constructed for each scenario; probability of rabies entry into north-west Cape York Peninsula (Scenario 1) and entry into Seisia (Scenario 2) *via* an individual Indonesian fishing boat; annual number of rabies-infected dogs and the probability of entry of at least one rabies-infected dog entering per year in north-west Cape York Peninsula (Scenario 1) and Seisia (Scenario 2); the probability of rabies entry and transmission (introduction) into Seisia (Scenario 2) *via* an individual Indonesian fishing boat; and finally, the probability of introduction from at least one rabies-infected dog per year in Seisia (Scenario 2). Also calculated is the time until next event for each scenario—the number of years for one rabies-infected dog to enter north-west Cape York Peninsula (Scenario 1) and Seisia (Scenario 2) as well as the number of years for one rabies-infected to dog to transmit rabies to a resident Seisia dog (Scenario 2). This is estimated by a gamma distribution using the respective number of infected dogs per year for each scenario.

### Expert Opinion Workshop

#### Overview

Following structured interviews and description of entry pathways, an expert opinion workshop was conducted to derive parameters to assess the risk of entry of rabies-infected dogs to north-west Cape York Peninsula and Seisia. Workshop participants were 13 NPA community members—7 Land and Sea Rangers, 3 Biosecurity Officers, a manager from the Northern Peninsular Area Regional Council, an Environmental Management Worker, and the manager of the local community enterprise business. The distribution of ages and expertise of participants are shown in Table 2. A modified Delphi technique and participatory methods (proportional piling) were used to elicit information from the participants.

**Table 2:** Expert opinion participant information from a workshop conducted in 2016 to parameterize a risk assessment of rabies entry *via* Indonesian fishing boats into north-west Cape York Peninsula and rabies introduction to a resident dog in Seisia, Queensland, Australia.

Participant occupation	Age	Sex	Years in current job
Biosecurity officer	20–30	Male	<1
Biosecurity officer	30–40	Male	1–7
Biosecurity officer	40–50	Male	8–15
Council manager	50–60	Female	1–7
Council manager	50–60	Male	8–15
Land and sea ranger	20–30	Female	<1
Land and sea ranger	18–20	Female	<1
Land and sea ranger	20–30	Male	<1
Land and sea ranger	30–40	Male	1–7
Land and sea ranger	20–30	Male	1–7
Land and sea ranger	20–30	Male	1–7
Land and sea ranger	30–40	Male	1–7
Local business manager	50–60	Male	1–7

#### Delphi Process

A four-step elicitation procedure was used to elicit values and limit overconfidence of estimates (20). Participants were asked to individually estimate the minimum, most likely, and maximum values as well their confidence that their minimum–maximum interval contained the true value. This procedure was used to gather information about the number of boats arriving at north-west Cape York Peninsula, the duration of travel from Indonesia, duration of stay in north-west Cape York Peninsula, and the numbers of dogs per boat. The experts’ intervals were standardized to 80% confidence intervals using Eqs 1 and 2, in which α_abs_ is the standardized minimum estimate, α is the expert’s minimum estimate, γ is the expert’s most likely estimate, β_abs_ is the standardized maximum estimate, β is the expert’s maximum estimate, *c* is the expert’s confidence level, and *p* is the standard confidence interval (19, 32). The distributions of expert opinions were combined to create empirical distributions for each parameter.

(1) αabs=γ−(γ−α)(cp).

(2) βabs=γ+(β−γ)(cp).

#### Proportional Piling

Proportional piling was used in group exercises in which participants openly discussed probabilities and counts associated with the scenarios and illustrated their opinions using piles of jellybeans (33). Participants used this method to demonstrate the numbers of boats that land at locations in Cape York Peninsula. They also used proportional piling to describe the minimum, most likely, and maximum values for the proportion of boats that carry dogs and the proportion of dogs that disembark (dependent on disease progression and whether dogs appeared sick or healthy). These values were used to create PERT distributions for each parameter.

### Sensitivity Analysis

Sensitivity analyses were performed using variance-based global sensitivity analysis and the Sobol method from the “sensitivity” package in R (34). We assessed one outcome for each scenario—the annual number of infected dogs from Indonesia that enter north-west Cape York Peninsula (*Scenario 1*) and the probability that a rabies-infected dog arrives *via* boat from Indonesia and infects a resident Seisia dog (*Scenario 2*) following the landing of an Indonesian boat in Seisia. The Sobol method estimates each input distribution’s influence on the variance of the outcome variable both individually (“main” or “first-order” effect) and with interactions with other inputs (“total” effect). Sensitivity indices (SIs) were estimated for each input’s main and total effects; the higher the SI, the more influence the distribution has on the outcome. Plots of total and main effect SIs for each scenario outcome were created with 95% confidence intervals estimated using the “boot” package in R (35, 36).

### Parameter Information

#### Annual Number of Boats Entering North-West Cape York Peninsula (Scenario 1) and Seisia (Scenario 2)

An empirical distribution of the number of boats arriving in north-west Cape York Peninsula was estimated using the modified Delphi technique to elicit values in the expert opinion workshop. A binomial distribution with the probability of a Seisia landing (determined using proportional piling) and the annual number of boats entering north-west Cape York Peninsula was used to describe the annual number of boats entering Seisia.

#### Number of Dogs Entering per Boat in North-West Cape York Peninsula (Scenario 1) and Seisia (Scenario 2)

The modified Delphi technique during the expert opinion workshop was used to derive the number of dogs per boat with at least one dog. Seisia is part of north-west Cape York Peninsula; therefore, the same distribution was used for boats entering Seisia (Scenario 2).

#### Node 1—Probability of a Dog on Board a Fishing Boat from Indonesia

The probability of at least one dog on board an Indonesian fishing boat that arrives in north-west Cape York Peninsula was determined in the expert opinion workshop using proportional piling. The minimum, most likely, and maximum values given were used to create a PERT distribution. This probability was also used for boats landing in Seisia community (*Scenario 2*).

#### Node 2—Probability That a Dog Is Infected with Rabies

The probability that a dog is infected with rabies depends on the prevalence of rabies at its origin in Indonesia. Rabies prevalence or incidence varies throughout endemic regions in Indonesia and is difficult to measure due to the potentially long incubation period of rabies and surveillance constraints. For example, the monthly attack rate of rabies in dogs (laboratory confirmed cases) in an outbreak in Bali was estimated to be 0.0003 prior to vaccination campaigns (4). Another study estimated a prevalence of 0.005 in Flores and 0.01 in South Sulawesi (22). Similarly low prevalence or incidence rates have been reported in other rabies endemic regions such as in Tanzania, Kenya, and Chad, where reported incidence of rabies in unvaccinated dogs has ranged between 0.0014 and 0.01 (24–26). Even in outbreak situations—such as that seen in Bhutan in the eastern districts in 2005–2007 and in the Chukha District in 2008—incidence can still be low (0.0143 and 0.02, respectively) (5, 6). Considering these low but varying estimates in regions around the world, a uniform distribution of 0.0001–0.05 was used in this risk assessment to describe the probability that a dog was infected with rabies.

#### Node 3—The Probability of Surviving Travel

The probability that the dog survives the journey and its stage of disease is dependent on the duration of travel from Indonesia and the length of incubation and clinical periods. The duration of travel was described by an empirical distribution derived from the expert opinion workshop. The minimum duration of travel was assumed to be no less than 1 day; therefore, the derived empirical distribution was truncated to reflect this. The incubation period depends on virus strain, the anatomical location of the bite, and virus dose. A study of 98 rabies cases in dogs from data collected in Tokyo, Japan, estimated a mean incubation period of 27 days (SD 20 days) (27). By using these data, we reproduced a lognormal distribution to describe the incubation period in this risk assessment (median 19 days; 95% percentile 5.4–68.6 days). A gamma distribution was used to describe the duration of the clinical period based on reconstructed case histories of over 1,000 suspected rabid dogs in Tanzania (28). We reproduced this distribution to describe the duration of clinical period in the risk assessment model (mean 3.1 days; 95% percentile 0.6–7.7 days).

The probability that a dog survived travel and its stage of infection on arrival was based on the following simulation. An incubation period, duration of travel, and duration of clinical signs were randomly selected from their respective distributions. The duration of disease was the combined incubation and clinical periods. We assumed that a dog showing clinical signs would not be taken aboard a boat in Indonesia. Therefore, the day of disease progression on which the dog started its journey to Australia was randomly chosen from the incubation period. The day of disease progression on arrival in Australia was calculated from the travel duration plus the randomly chosen start day. If the day of disease progression on arrival was greater than the duration of infection, the dog died during travel. If the day of disease progression on arrival was less than the incubation period, the dog arrived during the incubation period. If the day of disease progression on arrival was less than the duration of disease but greater than the incubation period, the dog arrived within the clinical period. The number of dogs in each category were counted, and a distribution for the probability of a dog arriving in each category was derived.

#### Node 4—Probability That a Rabies-Infected Dog Disembarks in Australia

This node describes the probability that a rabies-infected dog disembarks from the boat once it has arrived in north-west Cape York Peninsula. Workshop participants stated that the probability of disembarkation will be dependent on whether the dog is in the clinical phase or still incubating and not showing clinical signs. The duration of stay of the fishermen once arrived in north-west Cape York Peninsula could also affect this probability. For example, higher probabilities of disembarkation might be associated with longer duration of stay. The modified Delphi technique was used to derive the duration of stay in north-west Cape York Peninsula. Proportional piling during the workshop was used to estimate the probability of dog disembarkation if the dog was either showing or not showing clinical signs, for durations of stay of <5 days and ≥5 days (the 5-day threshold was defined by the participants of the workshop). The two probabilities for each disease stage were then combined by randomly selecting a value from the duration of stay distribution and then randomly selecting a value for the probability of disembarkation from the relevant probability distribution (either <5 or ≥5 days).

#### Node 5—Probability That a Rabies-Infected Indonesian Dog Meets a Resident Dog

The probability that a rabies-infected dog that arrives on an illegal fishing boat from Indonesia meets a resident NPA dog is dependent on the time that resident dogs are present on Seisia beach. We used the term “beach-minutes” to describe the number of minutes of each day that a resident dog could be expected to be on the Seisia beach. The total beach-minutes per day was estimated from previously collected GPS data of 29 dogs in Seisia [September 2013 (*n* = 9 dogs), April and September 2014 (*n* = 8 and 7 dogs, respectively) and June 2015 (*n* = 5 dogs)] for which the GPS fix interval was set at 1 min (37). From these datasets, the number of beach-minutes for each dog was estimated using a GPS coordinate error of 18 m. The median time-gap between GPS locations was added to each beach-trip to adjust for the time spent on the beach before and after the recorded GPS fixes. Poisson distributions were used to describe variability of each dog’s daily beach-minutes.

Of the 29 dogs collared, 20 dogs (68%) visited the beach at least once; therefore, we made the assumption that approximately two-thirds of the current population would spend time on the beach. The current population of dogs in Seisia is approximately 50 dogs (21). Therefore, 30 Poisson distributions were randomly selected (with replacement) and combined to simulate the daily beach-minutes of the dogs that visit the beach in Seisia. This was repeated 100 times, and the resulting distributions were combined to produce an overall distribution of daily beach-minutes. The probability that a resident dog would be on the beach when a rabies-infected dog arrives on an Indonesian fishing boat was described by a beta distribution derived from the overall beach-minutes distribution.

#### Node 6—Probability an Indonesian Dog Bites a Resident Dog

The probability that a clinically affected rabies-infected Indonesian dog bites a resident Seisia dog is likely to differ depending on whether the dog has developed the dumb or furious form of rabies. We assumed that the probability a dog with the dumb form of rabies bites another dog is likely to be similar to background dog bite incidence. By using data collected from a survey of 178 dogs in May 2016, a background probability of receiving a bite between NPA dogs was estimated as 0.00693 (38 dogs received a bite during the previous month). This probability is likely to be an underestimate due to recall bias (the owners surveyed did not remember all the dog bites) or lack of observation of minor bites. Also, these bites are between NPA dogs and do not account for the possibility that bite probability could be increased due to aggression between unfamiliar dogs. This estimate is also the probability of a dog receiving a bite—not the probability of a dog biting another dog—and so to account for these uncertainties a uniform distribution of 0.001–0.1 was used to describe the daily bite probability of a dog with the dumb form of rabies. If the dog has developed furious rabies, the probability that it bites another dog is likely to be higher. The probability of developing furious rabies is variable. Observational studies in which samples of suspect animals are sent for testing have suggested that high proportions (up to 0.75) of rabies-infected dogs develop furious rabies (31). However, these studies are likely to overestimate this proportion due to reporting bias (furious cases are more likely to be recognized and submitted for testing). Experimental testing has demonstrated a lower proportion (between 0.05 and 0.4) of furious rabies development (29, 30, 38). In the current study, the probability of developing furious rabies was therefore assumed to be a uniform distribution 0.1–0.6. The bite probability of a dog with the furious form is likely to be high due to the aggressive behavior associated with this form of rabies. Hampson et al. (28) estimated dog bites using a negative binomial distribution of mean 2.15 bites/rabid dog (95% CI 1.95–2.37) and clinical period 3.1 days (95% CI 2.9–3.4). This produces an approximate mean daily bite probability of 0.7. We assumed that most dogs in the study by Hampson et al. (28) developed the furious form because dogs suffering from the furious form would be more likely to be observed and therefore used to calculate bite probability. Therefore, we used a uniform distribution 0.5–0.8 around the approximate mean calculated from the study by Hampson et al. (28) to describe the probability of a bite if the dog developed furious rabies.

#### Node 7—Probability That a Resident Dog Develops Rabies following a Bite

Australia is canine rabies free; therefore, dogs are unvaccinated. There is natural variability in the probability of rabies development after a dog bite, dependent on virus strain, the anatomical location of the bite, and virus dose. Hampson et al. (28) estimated that the probability that an unvaccinated dog would develop rabies following a bite from a rabies-infected animal was 0.49 (95% CI 0.45–0.52). It is possible that severe bites were more likely observed. The probability of rabies transmission might be lower following a less severe bite in which less virus is transferred. Therefore, a uniform distribution of 0.35–0.52 was used to describe the probability of rabies infection following a bite from a rabid animal.

## Results

### Parameter Information

All distributions used in the model are described in Table 1.

#### Number of Boats Arriving in the North-West Cape York Peninsula (Scenario 1) and Seisia (Scenario 2) per Year

**Figure 4:**
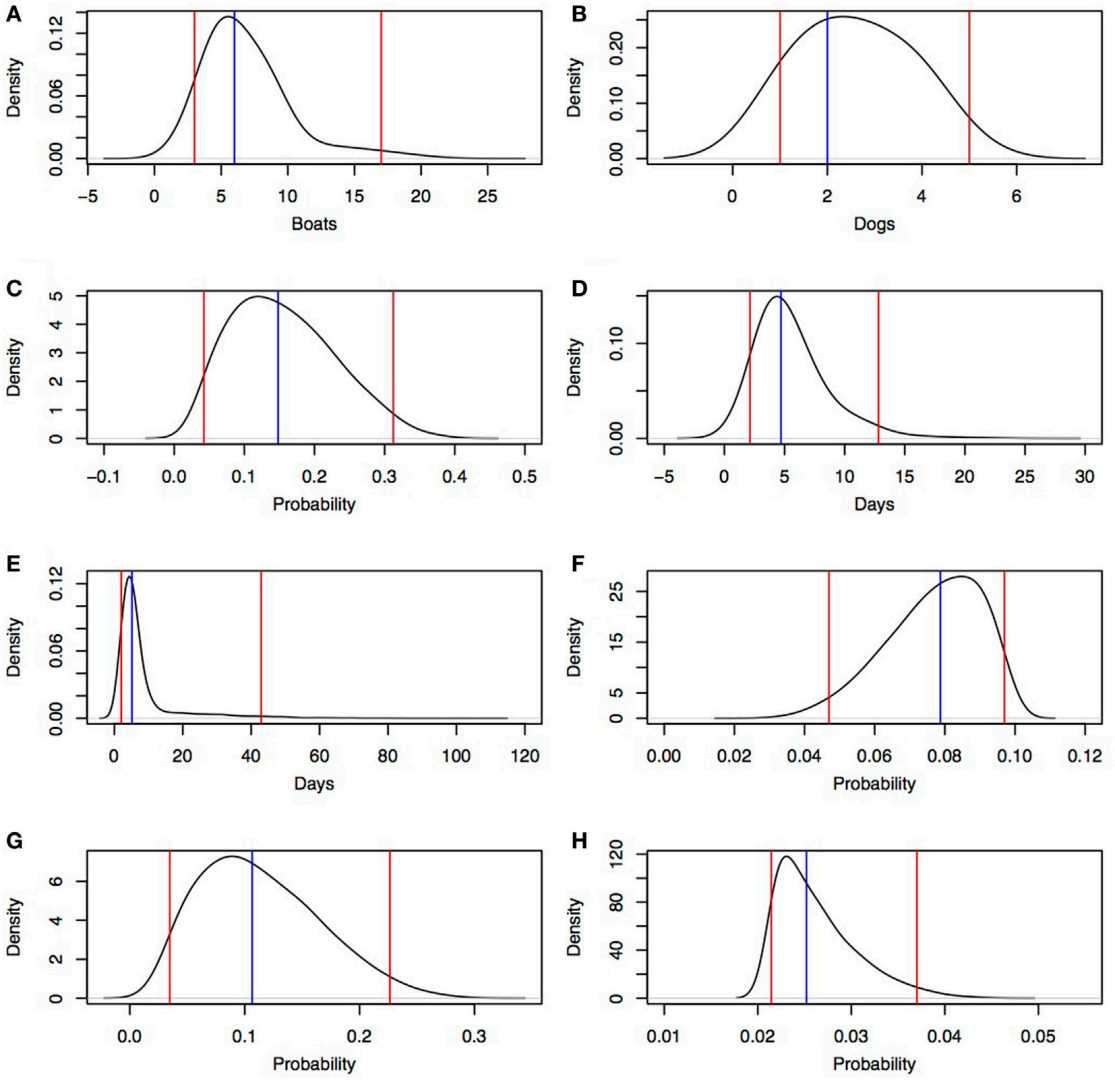
Density distributions derived from an expert opinion workshop in a rabies risk assessment, north-west Cape York Peninsula, Australia. Blue lines, median; red lines, 95% percentile. **(A)** number of boats arriving at north-west Cape York Peninsula per year, **(B)** number of dogs per boat for boats with at least one dog, **(C)** probability an Indonesian fishing boat has at least one dog, **(D)** duration of travel from Indonesia to north-west Cape York Peninsula, **(E)** duration of stay of Indonesian fishermen after landing, **(F)** probability an apparently healthy dog disembarks if staying <5 days, **(G)** probability an apparently healthy dog disembarks if staying ≥5 days, and **(H)** probability a clinically affected dog disembarks.

The median number of Indonesian fishing boats (regardless of whether a dog is on board) arriving in north-west Cape York Peninsula per year was estimated to be 6 (95% percentile 3–17 boats) based on the combined individual PERT distributions from workshop information (Figure 4A). Workshop participants estimated that the proportion of the boats that traveled to north-west Cape York Peninsula and landed in Seisia was 0.0455. Therefore, the median number of Indonesian fishing boats landing in Seisia was 0 boats per year (95% percentile 0–2 boats).

#### Number of Dogs per Boat Arriving in North-West Cape York Peninsula (Scenario 1) and Seisia (Scenario 2)

The median number of dogs on boats with at least one dog was 2 dogs per boat (95% percentile 1–5 dogs/boat; Figure 4B).

#### Node 1—Probability of at Least One Dog on Board a Fishing Boat from Indonesia

Estimates generated by the workshop participants (0.02, 0.12, and 0.43 for minimum, most likely, and maximum probabilities, respectively, of at least one dog on board a fishing boat from Indonesia) were used to create a PERT distribution (Figure 4C). Participants stated that variability in these probabilities depended on whether the fishermen owned a dog and the secondary activities of the fisherman once they arrived in Australia (for example, if hunting was expected, they were more likely to bring dogs).

#### Node 3—Probability of Stage of Rabies Infection on Arrival

The median duration of travel from Indonesia to north-west Cape York Peninsula was 4.7 days (95% percentile 2.1–12.8 days; Figure 4D). Workshop participants noted that duration of travel is dependent on origin in Indonesia and weather conditions. The simulation produced three probabilities; two probabilities for a dog that has survived the journey to north-west Cape York Peninsula [one for each arrival disease state—incubating (not showing clinical signs)] and clinical (showing clinical signs) and one for the probability the dog died from rabies during travel. The most likely outcome was that a dog arrives during its incubation period (median 0.65; 95% percentile 0.64–0.66); lower probabilities were estimated for a dog dying of rabies during travel (median 0.18; 95% percentile 0.17–0.18) and a dog arriving during its clinical period (median 0.17; 95% percentile 0.16–0.17).

#### Node 4—Probability That a Rabies-Infected Dog Disembarks in Australia

The empirical distribution from the expert opinion workshop describing duration of stay of the fisherman had a median of 5.2 days (95% percentile 2.1–42.9 days; Figure 4E). Participants noted that the duration of stay of fisherman in north-west Cape York Peninsula was influenced by success of fishing; knowledge of Australian Border Force operations; or presence of local relatives, trade contacts, and friends. The probability that an apparently healthy (incubating) dog disembarked was higher than the probability that a clinically affected dog disembarked because a dog showing overt clinical signs was believed to be more likely to be killed during travel or left on the boat on arrival and, therefore, less likely to disembark. In addition, participants noted that if the fishermen stayed ≥5 days, the probability that an apparently healthy dog would disembark could be greater than if the fishermen stayed <5 days. Therefore, two PERT distributions were parameterized from values given in the workshop with a higher maximum value if fishermen stayed ≥ 5 days (most likely 0.0851, minimum 0.0213, and maximum 0.3191; most likely 0.0851, minimum 0.0213, and maximum 0.1, respectively; Figures 4F,G). The combined probability that an incubating dog disembarked had a median of 0.085 (95% percentile 0.039–0.207). The participants stated that duration of stay did not affect the probability that a clinically affected dog disembarked. Therefore, only one PERT distribution was derived from the expert opinion—regardless of duration of stay—to describe the probability of disembarkation of a clinically affected dog (most likely, minimum, and maximum estimates of 0.021, 0.021, and 0.051, respectively; Figure 4H).

#### Node 5—Probability That a Rabies-Infected Indonesian Dog Contacts a Resident Dog (Scenario 2)

**Figure 5:**
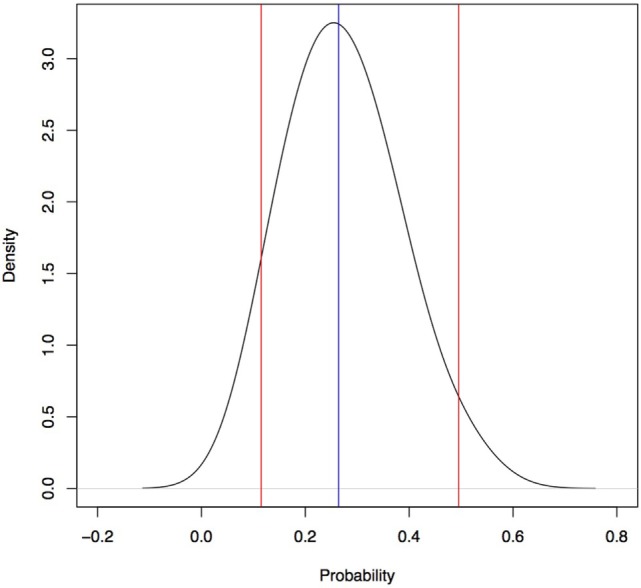
Daily probability a dog is on the beach at Seisia used in a rabies risk assessment, north-west Cape York Peninsula, Australia. Blue line, median; red lines, 95% percentile.

The estimated median number of daily beach-minutes that a resident dog would be on Seisia beach was 379 minutes/day (95% percentile 171–719 minutes/day). The estimated median daily probability that a Seisia dog will be on the beach at the time an Indonesian dog disembarks was 0.26 (95% percentile 0.12–0.50; Figure 5).

### Model Outputs

#### Probability of Rabies Entry into North-West Cape York Peninsula (Scenario 1) and Entry into Seisia (Scenario 2) *via* an Individual Indonesian Fishing Boat

**Figure 6:**
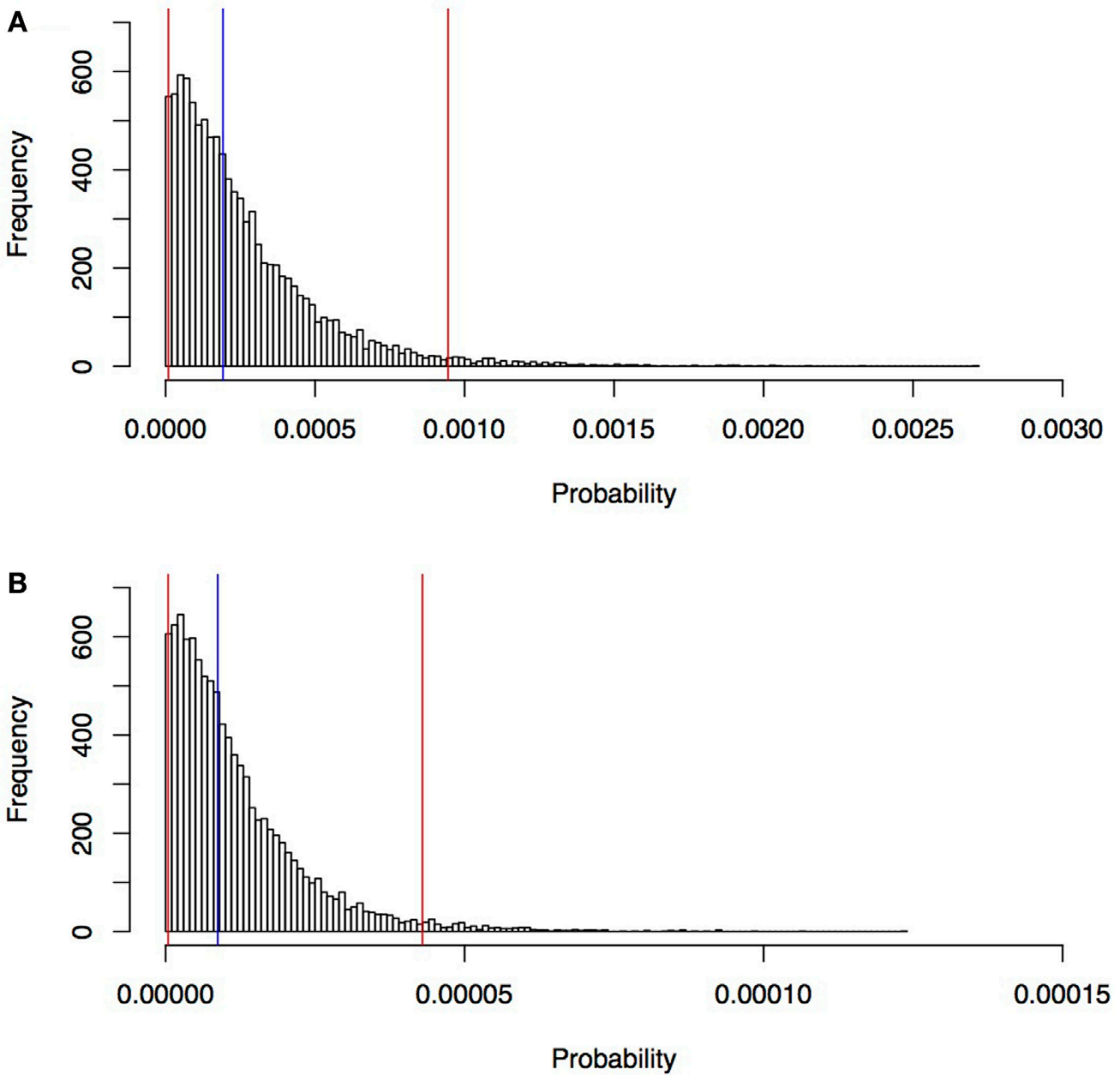
Estimated probability of rabies entry into north-west Cape York Peninsula, Queensland **(A)**, and into Seisia **(B)** *via* an individual Indonesian fishing boat estimated in a rabies risk assessment, north-west Cape York Peninsula, Australia. Blue line, median; red lines, 95% percentile.

The estimated median probability of rabies entry into north-west Cape York Peninsula (*Scenario 1*) *via* an individual Indonesian fishing boat landing was 1.9 × 10^−4^/boat (95% percentile 9.3 × 10^−6^–9.4 × 10^−4^/boat) and 8.7 × 10^−6^ (95% percentile 4.2 × 10^−7^–4.3 × 10^−5^/boat) for entry into Seisia (*Scenario 2)*. These distributions are shown in Figure 6.

#### Annual Number of Rabies-Infected Dogs and the Probability of Entry of at Least One Rabies-Infected Dog Entering per Year in North-West Cape York Peninsula (Scenario 1) and Seisia (Scenario 2)

The estimated annual median number of dogs that arrive by boat in north-west Cape York Peninsula was 2 (95% percentile 0–11 dogs), of which the median number of rabies-infected dogs was 0 dogs (95% range 0–0 dogs; maximum 2 dogs). The estimated probability that at least one rabies-infected dog is introduced per year was 5.5 × 10^−3^ (SE 1.0 × 10^−3^). The estimated probability that at least one dog in the incubation period of rabies is introduced per year (3.1 × 10^−3^; SE 9.3 × 10^−4^) was higher than the probability that at least one dog in the clinical period of rabies is introduced per year (6.4 × 10^−4^; SE 3.4 × 10^−4^). The estimated median time between events of one rabies-infected dog entering north-west Cape York Peninsula is 135.3 years (95% percentile 4.9–715.9 years).

The estimated annual median number of dogs entering Seisia by boat per year was 0 (95% percentile 0–2 dogs), of which the estimated median number of rabies-infected dogs that enter Seisia per year was 0 dogs (95% range 0–0 dogs; maximum 1 dog). The estimated probability that at least one rabies-infected dog is introduced per year was 3.5 × 10^−4^ (SE 2.8 × 10^−4^). The estimated probability that at least one dog in the incubation period of rabies is introduced per year (2.4 × 10^−4^; SE 2.4 × 10^−4^) was higher than the probability that at least one dog in the clinical period of rabies is introduced per year (9.9 × 10^−5^; SE 1.4 × 10^−4^). The estimated median time between events of one rabies-infected dog entering Seisia is 1,724.0 years (95% percentile 59.5–9,381.2 years).

#### Introduction of Rabies to Seisia (Scenario 2)

**Figure 7:**
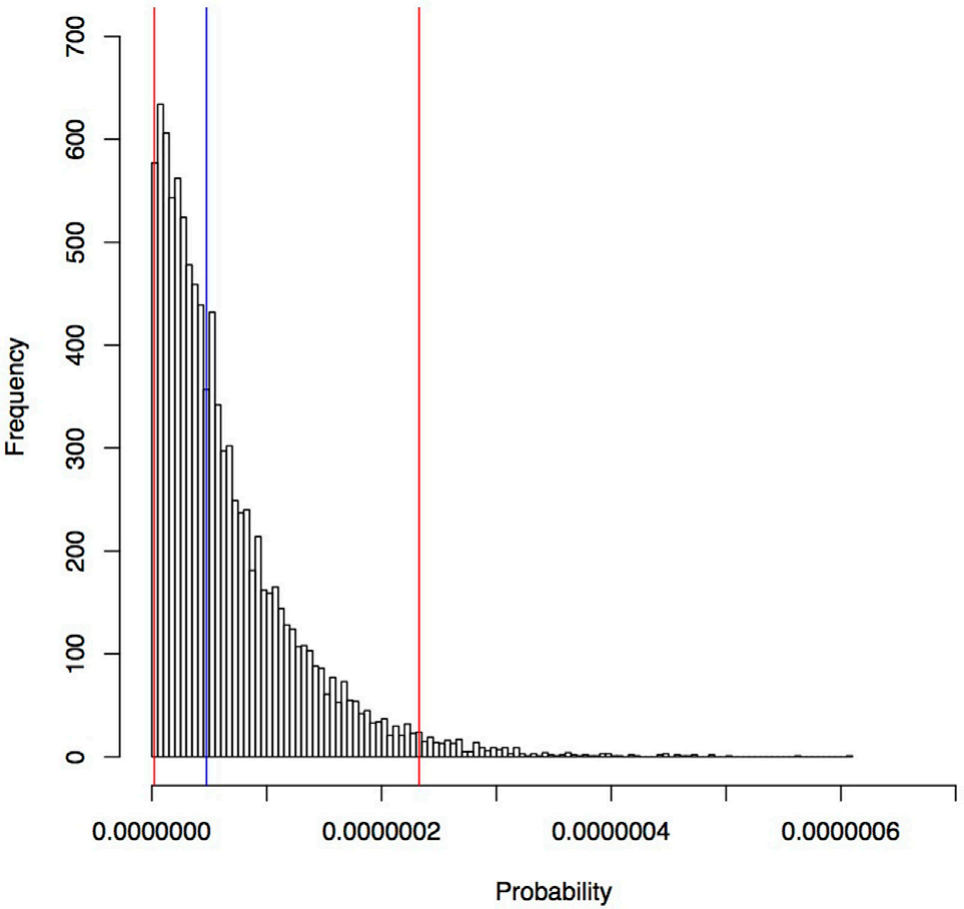
Estimated probability of rabies introduction into Seisia *via* an individual Indonesian fishing boat estimated in a rabies risk assessment, north-west Cape York Peninsula, Australia. Blue line, median; red lines, 95% percentile.

The estimated median probability of rabies introduction into Seisia *via* an individual Indonesian fishing boat landing was 4.7 × 10^−8^/boat (95% percentile 2.1 × 10^−9^–2.3 × 10^−7^/boat) and is shown in Figure 7. The estimated probability that at least one rabies-infected dog from an Indonesian fishing boat transmits rabies to a Seisia dog per year was 8.3 × 10^−5^ (SE 1.4 × 10^−4^). The estimated median time between events of one rabies-infected dog entering and exposing rabies to a Seisia dog is 6,777.0 years (95% percentile 247.2–36,368.8 years).

### Sensitivity Analysis

**Figure 8:**
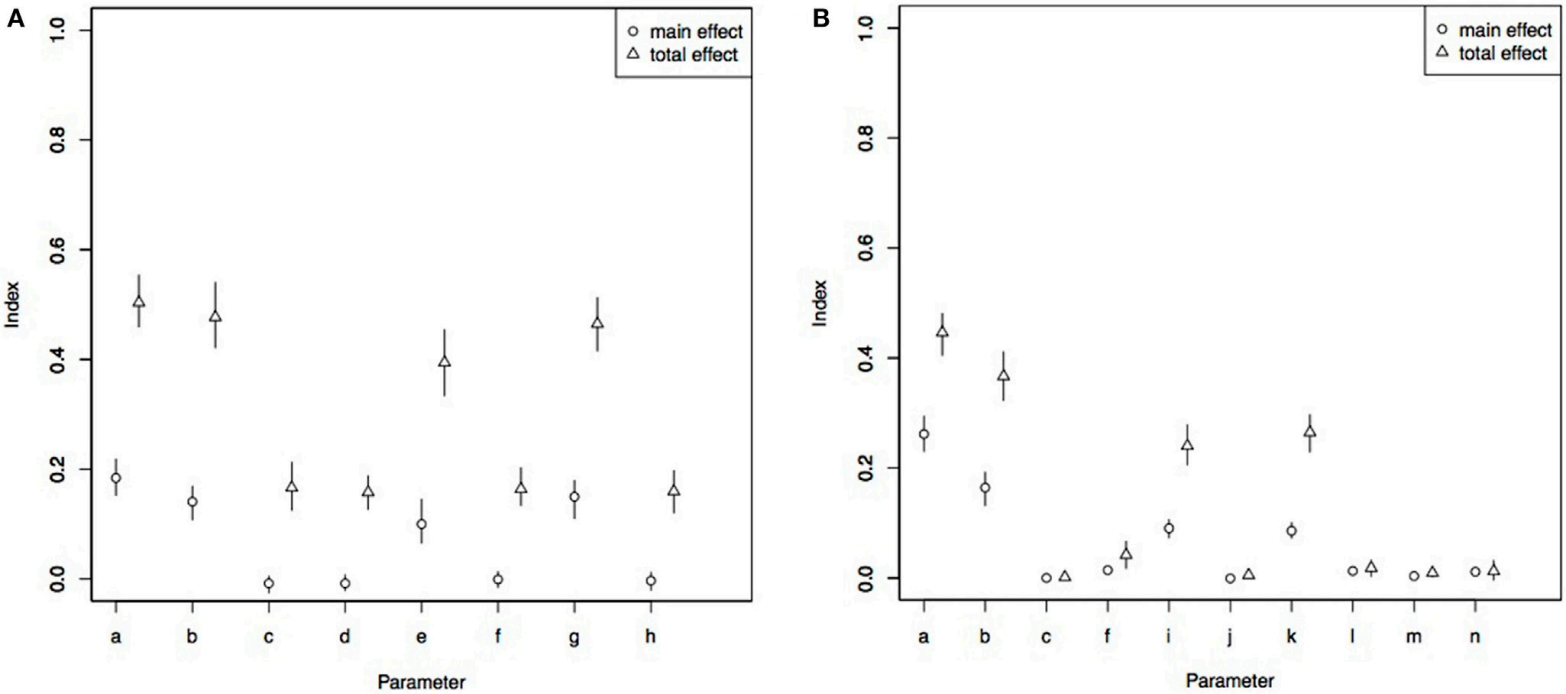
Total and main effect sensitivity index from Sobol sensitivity analysis in a rabies risk assessment, north-west Cape York Peninsula, Australia, for number of boats likely to bring a rabies-infected dog to north-west Cape York Peninsula per year **(A)** and probability of rabies introduction into Seisia **(B)**. a, prevalence of rabies in Indonesia; b, probability of at least one dog on board an Indonesian fishing boat; c, probability dogs arrives in clinical period; d, probability dog arrives in incubation period; e, probability apparently healthy dog disembarks; f, probability clinically affected dog disembarks; g, number of boats per year arriving in north-west Cape York Peninsula; h, number of dogs per boat; i, probability clinically affected dog develops furious rabies; j, probability clinically affected dog develops dumb rabies; k, daily probability a Seisia dog is on the beach in Seisia; l, probability a clinically affected (furious) Indonesian dog bites a Seisia dog; m, probability a clinically affected (dumb) Indonesian dog or rabies-infected Indonesian dog in the incubation period bites a Seisia dog; n, probability Seisia dog develops rabies following bite.

The most influential parameter on the number of infected dogs from Indonesia that enter north-west Cape York Peninsula was prevalence of rabies in Indonesia (total effect SI 0.50) followed by the probability of a dog on board a boat (total effect SI 0.48), the number of boats arriving in north-west Cape York Peninsula (total effect SI 0.46) and probability of an apparently healthy dog disembarking (total effect SI 0.39). The other inputs were less influential (total effect SI <0.25). Total effect SIs were higher than main effect SIs for all influential parameters. The total and main effect SIs are shown in Figure 8.

The most influential parameter for the probability of rabies introduction to a resident dog in Seisia was the prevalence of rabies in Indonesia (total effect SI 0.44) followed by the probability a dog is on board the boat (total effect SI 0.36), the probability of a Seisia dog on the beach (total effect SI 0.26), and the probability the infected dog developed the furious form of rabies (total effect SI 0.23). Total effect SIs were higher than main effect SIs for all influential parameters. The total and main effect SIs are shown in Figure 8.

## Discussion

Although the probabilities of entry of a rabies-infected dog *via* an illegal Indonesian fishing boat into north-west Cape York Peninsula, Queensland, or the subsequent transmission to a resident dog in Seisia in this region are low, the impact of a rabies incursion into this region are potentially large. Therefore, the overall risk is not negligible. Sensitivity analysis highlighted inputs that could be targeted for further data collection to refine model outputs or surveillance and control to mitigate rabies incursion risk.

The prevalence of rabies in Indonesia was the most influential parameter on both the entry of rabies into north-west Cape York Peninsula and introduction to a resident dog in Seisia. Prevalence of rabies in the region of origin has also been found influential in other risk assessments (14, 22, 23). The wide distribution given to this parameter reflects the variability of rabies prevalence in Indonesia and globally and the uncertainty of rabies prevalence in Indonesia due to limited surveillance. Its importance to the outcome suggests further surveillance for refinement of the model parameter and surveillance and control to help reduce rabies prevalence in Indonesia as a pre-border control for Australia. Given its high importance, vaccination campaigns to limit rabies prevalence in Indonesia would be the most effective measure to limit rabies risk of entry and transmission into northern Australian communities like the NPA compared to domestic control measures.

Some expert opinion-derived parameters had high influence on outcomes. The distributions for these parameters were wide. Although the workshop participants were targeted to achieve a sample of people with knowledge of Indonesian boat arrival into north-west Cape York Peninsula, and the structured format of the modified Delphi process was used to reduce cognitive bias (20), these wide distributions are likely to reflect uncertainty about some parameters. This also reflects the lack of documented data about these parameters. Empirical data—such as coastline surveys or surveys conducted at ports and informal ports—would complement the expert opinion derived in this workshop. Port surveys have been conducted in Japan and in Lombok, Indonesia, and provided empirical evidence regarding the probability of a dog on board a boat, the number of dogs per boat, and the number of boats arriving from a certain origin (22, 23). Of the influential parameters to mitigate rabies risk, the number of boats arriving at Cape York Peninsula is perhaps the most feasible to control. Rabies entry and transmission risk through illegal Russian boat landings has been successfully reduced in Japan by limiting the number of boats entering Hokkaido *via* education of Russian fishermen, establishment of warning signs, daily patrols, and regular port surveillance (23). This could be beneficial for limiting the number of boats that land specifically in Seisia. However, intensive surveillance along the entirety of the coastline of north-west Cape York Peninsula would be costly. Most of the Indonesian fishing boats are landing in the sparsely populated areas of north-west Cape York Peninsula, and so the cost of surveillance would need to be balanced with the frequency of surveillance surveys required to reach an appropriate level of protection.

The inclusion of assessment of transmission to a resident Seisia dog in this study was beneficial because it highlighted further aspects of the risk pathway that could be targeted for surveillance and control for risk mitigation—the probability of meeting a Seisia dog on the beach. This differs from the study by Kwan et al. (23) who found the probability of contact with resident dogs (stray or owned) was not highly influential on the outcome; instead, the probability of contact with wildlife was influential. This difference might be due to different dog owning cultures. Most dog owners in Hokkaido keep their dogs on leads, and effective communication of rabies exposure risk has caused owners to avoid the ports. This limits exposure and subsequent transmission opportunities. In contrast, dog owners in Seisia allow their dogs to freely roam giving dogs access to the beach and therefore, a higher chance of contact with an infected dog. The probability of meeting a Seisia dog on the beach is a factor that can be controlled by limiting the resident dogs’ access to Seisia beach. However, this has logistic challenges. A previous survey conducted in the NPA in 2016 showed that there was high willingness to confine domestic dogs to residents’ homes by closing yard fences, chaining dogs, or keeping them inside (39). However, this was in the context of a disease outbreak and focused on short-term outbreak control strategies only. Also, confinement in yards is limited due to a lack of fencing sufficient to effectively confine dogs—many residents stated that their dogs were capable of escaping a closed yard (39). The probability of development of the furious form of rabies in clinically infected Indonesian dogs was also influential on the outcome for Scenario 2. The wide distribution given to this parameter was to reflect the high variability seen in published papers and potential uncertainty of the probability. Targeted surveillance and further data collection—including field studies of infected dogs in rabies-infected regions—could help refine this parameter and model outputs.

Pre-emptive vaccination of resident Seisia dogs against rabies could also reduce the susceptible population on the beach in Seisia and therefore, the probability of effective contact. Currently, very few rabies-free regions adopt mandatory rabies vaccination, for example, Japan and Hong Kong. Some countries—such as Malaysia—have adopted a vaccine buffer zone along shared borders with rabies-infected countries as a preventive approach (3). The scientific justification for these policies in Japan has been questioned because a change in vaccination rate and efficacy did not significantly affect the risk of a rabies introduction in a risk assessment model, even when vaccination rate was reduced to zero (23). However, there are different exposure contexts between Seisia and Hokkaido. Most dogs around the Hokkaido ports are kept on leads (23). Conversely, in Seisia, most dogs are free roaming without leads or owner supervision (39), which leads to a higher probability of contact with a foreign rabies-infected dog. Also, the population with the greatest influence on risk of exposure in Hokkaido was wildlife not domestic dogs, which could also affect the influence of domestic dog vaccination in their study (23). Further research into the effects of a pre-emptive vaccination program on the reduction of rabies entry and exposure risk in Seisia could be used to investigate the benefits of a pre-emptive vaccination program in the NPA.

This study did not consider the exposure risk of wild-living dogs (domestic dogs and dingoes), which could lead to underestimation of the probability of rabies transmission in this study. Wild-living dogs inhabit most of Australia (40), including the sparsely populated north-west Cape York Peninsula and are known to frequent NPA communities (41). However, the lack of data on their abundance and distribution limits inclusion of wild-living dog transmission in a risk assessment for this region. Also, the current study focused on domestic dogs in Seisia due to the risk that this poses to public health. Inclusion of the probability of transmission to wild-living dogs in a future risk assessment of rabies in north-west Cape York Peninsula (including Seisia) would be beneficial for a more robust rabies risk assessment in which—similar to the risk assessment for rabies introduction into Hokkaido, Japan—controlling exposure and transmission into the these wild populations could be more influential than controlling exposure and transmission in the domestic dogs in north-west Cape York Peninsula.

There are limitations associated with the current study. We did not differentiate variance of model input and outputs into uncertainty and natural variability, due to insufficient empirical data about most of the parameters. A two-dimensional risk assessment in which uncertainty and variability are differentiated can aid interpretation of the sensitivity analysis. For example, if the variance of influential parameters is mainly due to uncertainty, more research to identify natural variability and its influence on risk is likely to be beneficial before recommendations for control measures are made. However, if the variance of influential parameters is mainly due to natural variability, recommendations for control measures that change the circumstances (for example, reducing number of dogs available for exposure and transmission) are likely to reduce risk. Other limitations arise from the analysis of GPS coordinate field data to estimate the daily probability a Seisia dog would be on the beach at the time of arrival of the Indonesian dog. This analysis did not consider that more than one dog could be present on the beach at any one point in time because each dog was considered separately. This could either underestimate the probability of contact (a greater number of dogs on the beach is likely to increase the opportunity for contact) or overestimate the probability of contact (overlapping minutes were counted twice, and consequently, the number of beach-minutes/day was higher). Also, the dogs that were GPS tracked were only tracked for short periods (median 3 days; range 1–13 days). This could underestimate the number of dogs that visit the beach, and subsequently probability of contact, because dogs that usually visit the beach may not have visited during GPS tracking. This was an influential parameter on the probability of rabies entry and exposure into Seisia; therefore, further surveys on dog roaming patterns along the beach could refine model outputs to produce a more accurate assessment of the risk of transmission of rabies to a resident Seisia dog. We also made the assumption that an Indonesian fishing boat would leave Seisia beach and take the dog with it. This could underestimate the probability of entry and introduction because for longer durations of stay or if the dog remained indefinitely in Seisia, the probability that a dog arriving during the incubation phase becomes clinical also increases, therefore increasing the number of dogs available to infect resident Seisia dogs. Also, the possibility that dogs could die during the incubation period of other causes—thus reducing the number of dogs arriving and disembarking—was not accounted for in the current model, which could consequently overestimate the risk of rabies entry. The assumption only clinically infected dogs will transmit rabies could also slightly underestimate the risk of rabies transmission into Seisia. In some cases, there is a short preclinical infectious period in which a dog is infectious but does not show clinical signs, which would increase the number of dogs capable of transmitting rabies and therefore the probability of transmission. This is unlikely to affect the overall results because it is generally very short leading to only a few more dogs being infectious, and the model was not sensitive to the probability of a dog arriving in the incubation or clinical stage of infection.

## Conclusion

This study revealed a low but not negligible risk of rabies entry into north-west Cape York Peninsula, Queensland, and introduction to a resident dog in Seisia *via* the transport of an infected dog on an Indonesian fishing boat. The most influential input in both sensitivity analyses was the prevalence of rabies entry and exposure in Seisia was the prevalence of rabies in Indonesia, which is a reflection of the uncertainty and variability of this parameter. Further data on the prevalence as well as other influential parameters such as the number of boats arriving and the probability of a dog on board would improve accuracy of model outputs. Furthermore, limiting the risk of rabies exposure could be achieved by limiting Seisia dogs’ access to the beach or reducing the susceptible population by vaccination.

## Ethics Statement

The protocol was approved and subsequently carried out in accordance to recommendations given by the Human Research Ethics Committee of The University of Sydney (reference number 2016/711) with written informed consent from all participants.

## Author Contributions

The study design, data collection, and manuscript writing and revision were completed by all authors (EH, VB, and MW). Data analysis and interpretation were completed by EH with contributions from VB and MW.

## Conflict of Interest Statement

The authors declare that the research was conducted in the absence of any commercial or financial relationships that could be construed as a potential conflict of interest.
